# Risk prediction models for mortality in patients with multimorbidity: a systematic review and meta-analysis

**DOI:** 10.3389/fpubh.2025.1505541

**Published:** 2025-04-02

**Authors:** Yuan-yuan Chen, Mei-fen Ji, Li-hong Jin, Lu-ga Dong, Min-hua Chen, Xu-li Shang, Xiang Lan, Yuan-yuan He

**Affiliations:** ^1^Department of Otorhinolaryngology, Organization Lishui People’s Hospital, Lishui, Zhejiang, China; ^2^Medical College, Organization Zhejiang University, Hangzhou, Zhejiang, China; ^3^Department of Nursing, Organization Lishui People’s Hospital, Lishui, Zhejiang, China; ^4^Department of Radiation Oncology, Organization Lishui People’s Hospital, Lishui, Zhejiang, China

**Keywords:** mortality, risk prediction model, systematic review, meta-analysis, multimorbidity

## Abstract

**Background:**

Multimorbidity is a major aging and public health problem that has a significant burden on a global scale. The number of risk prediction models for mortality in patients with multimorbidity is increasing; however, the quality and applicability of these prediction models in clinical practice and future research remain uncertain.

**Objective:**

To systematically review published studies on risk prediction models for mortality in patients with multimorbidity.

**Methods:**

The Wanfang, China National Knowledge Infrastructure, China Science and Technology Journal (VIP), PubMed, SinoMed, Cochrane Library, Web of Science, Embase, and Cumulative Index to Nursing and Allied Health Literature databases were searched from inception until May 30, 2024. Two independent reviewers performed study selection, data extraction, and quality assessment. The Prediction Model Risk of Bias Assessment Tool (PROBAST) checklist was utilized to assess the risk of bias and applicability.

**Results:**

Overall, 18 studies with 21 prediction models were included in this review. Logistic regression was used for model development in 12 studies, Cox regression in four, a parametric Weibull regression in one, and machine learning in one study. The incidence of mortality in patients with multimorbidity ranged from 7.6–50.0%. The most frequently used predictors were age and body mass index. The reported area under the receiver operating characteristic curve (AUC) and C-index values ranged from 0.700–0.907. Three studies were rated as having a low risk of bias, 11 as high, and four as unclear, primarily owing to poor reporting of the analysis domain. The pooled AUC value of the seven validated models was 0.81, with a 95% confidence interval ranging from 0.77–0.86, signifying a fair level of discrimination.

**Conclusion:**

The included studies revealed a degree of discriminatory ability in predicting mortality in patients with multimorbidity; however, they all demonstrated significant risks of bias based on the PROBAST checklist assessment. Future researchers should prioritize the development of new models that incorporate rigorous study designs and multicenter external validation, which may improve the precision of risk predictions and help the development of global strategies for this significant public health problem.

**Registration:**

The study protocol was registered in PROSPERO (registration number: CRD42024543170).

**Systematic review registration:**

https://www.crd.york.ac.uk/PROSPERO/recorddashboard, PROSPERO CRD42024543170.

## 1 Introduction

Multimorbidity is defined as the coexistence of two or more chronic conditions in the same individual and is frequently observed in medical and health research (1). The concept of comorbidity is related to that of multimorbidity (2) as comorbidity refers to any ailment that coexists with an index disease (3). Multimorbidity is estimated to affect a large proportion of the world’s population and demonstrates an S-shaped relationship with aging (4). The overall incidence of multimorbidity is 37.2%, and its highest incidence occurred in South America (45.7%), followed by North America (43.1%) and Asia (35.0%). More than half of the global adult population older than 60 years (51.0%) are suffering from multimorbidity. Multimorbidity has become increasingly prevalent in the last two decades, although the global incidence appears to have remained stable among adults in the recent decade (5). Multimorbidity has been associated with functional dependence, reduced productivity, poor quality of life, and increased mortality, threatening the sustainability of healthcare systems (6, 7). This represents a persistent and significant burden on individuals, families, healthcare systems, and societies. However, to the best of our knowledge, numerous evidence gaps exist regarding the relationship between multimorbidity and mortality. For instance, cancer and vascular disease are the two leading causes of mortality; however, the impact of multimorbidity on these disease outcomes remains unexplored (8). Multimorbidity is linked to a high mortality rate (9), and previous research has indicated that individuals with multimorbidity have an all-cause mortality risk that is approximately 2–3 times higher than that of those without multimorbidity (10–12). Nevertheless, it is potentially preventable (4) and easily preventable among individuals without chronic illnesses. For individuals diagnosed with a chronic illness, additional diagnoses are likely, and these linkages have been theorized to be mediated via sleep disturbance and circadian rhythm dysfunction (13). Multiple guidelines suggest tailoring the preventive care of individuals with multimorbidity based on life expectancy and consequently, their mortality risk (14, 15). Indeed, patients with a high short-term mortality risk, such as those with multimorbidity, may not benefit from preventive care interventions. Considering the rapid increase in the incidence of multimorbidity and the fact that the specific roles of various long-term conditions and their interactions in predicting mortality risk remain unclear, establishing a valid index for predicting mortality in patients with multimorbidity is necessary. The accurate prediction of mortality risk in such patients is crucial, as it can assist medical decision-making and enable health professionals to provide individualized treatments based on patients’ condition, orientation, and prognosis (16), and it is essential for early identification and intervention.

Prognostic models are mathematical equations incorporating multiple variables that can be used to estimate the probability that an individual in a specific health state will experience a particular health outcome (17). In this context, modeling the mortality risk for patients with multimorbidity to accurately estimate their individual risk has garnered increasing interest. Specifically, establishing risk of mortality models for patients with multimorbidity have been considered as the solution to many research problems. However, studies have found that the risk of mortality is differentially distributed among different comorbidity types (18, 19). Prediction models can assist healthcare providers in optimizing decisions, including accurately and rapidly identifying patients who require extensive education and preventive measures (e.g., weight control and regular monitoring); moreover, these models inform patients with multimorbidity and their family members on the mortality risk, enhancing their awareness and facilitating compliance with prevention (20, 21).

In the past decade, an increasing number of studies have been conducted to develop and validate risk prediction models for mortality in patients with multimorbidity (22, 23). However, a recognized and authoritative prediction model recommended for use by guidelines has not been established. Moreover, these models have been developed using small cohorts, lack external validation, and have rarely been applied in clinical practice (24), and the methodological quality has rarely been thoroughly and critically assessed. New models have been published; however, this problem remains unsolved. Hence, this systematic review aimed to screen and systematically review published studies on existing risk prediction models (developed or validated) for mortality in patients with multimorbidity.

## 2 Methods

### 2.1 Study design

This systematic review was conducted in accordance with the Preferred Reporting Items for Systematic Reviews and Meta-Analyses (PRISMA) 2020 guidelines. The study protocol was registered in PROSPERO (registration number: CRD42024543170).

### 2.2 Search strategy

**Figure 1:**
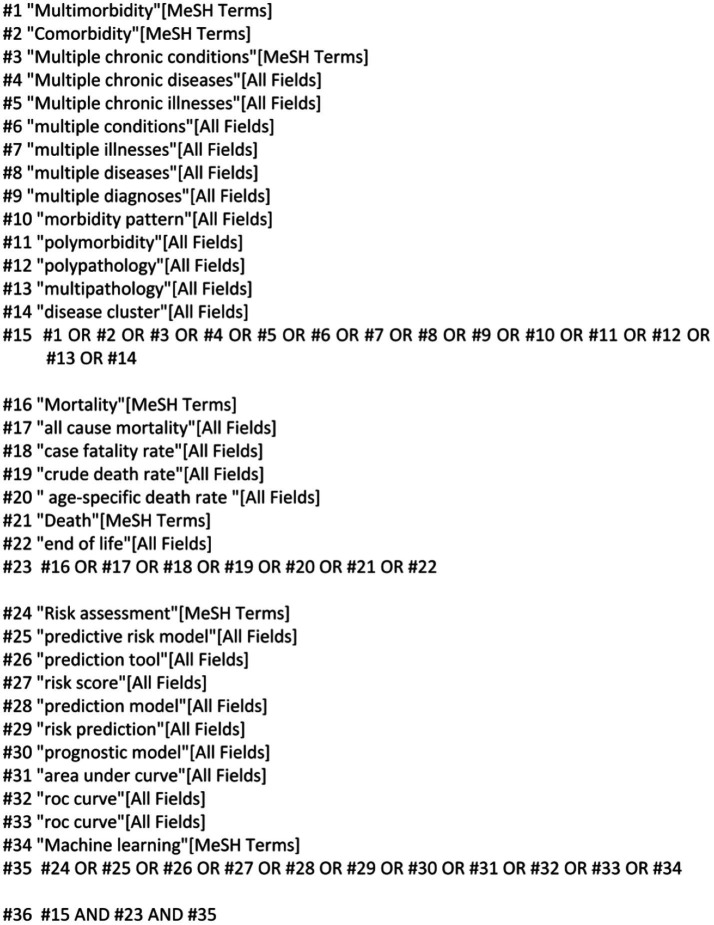
Search strategy.

Chinese and English databases were targeted to conduct a comprehensive search, considering the large population size and language universality. The Wanfang, China National Knowledge Infrastructure (CNKI), China Science and Technology Journal (VIP), PubMed, SinoMed, Cochrane Library, Web of Science, Embase and Cumulative Index to Nursing and Allied Health Literature (CINAHL) databases were searched from inception until May 30, 2024, using the following keywords: “multimorbidity,” “comorbidity,” “polymorbidity,” “mortality,” “death,” “case fatality rate,” “Risk prediction model,” “Risk factor,” “Predictor,” “Model,” and “Risk Score.” The retrieval method, using PubMed as an example, is shown in Figure 1. Additional relevant studies were identified by reviewing the reference lists of the retrieved studies and review articles.

We used the PICOTS system for the systematic review, which allows framing of the review’s aim, search strategy, and study inclusion and exclusion criteria, as described below.

P (Population): Patients with multimorbidity.

I (Intervention model): Risk prediction models for mortality in patients with multimorbidity that were developed and published (prediction score ≥ 2).

C (Comparator): No competing model.

O (Outcome): The outcome focused on mortality, rather than its subgroups.

T (Timing): The outcome was predicted after evaluating basic information upon admission, clinical scoring scale results, and laboratory indicators.

S (Setting): The intended use of the risk prediction models was to individualize the prediction of mortality risk in patients with multimorbidity, thereby facilitating the implementation of preventive measures for adverse events.

### 2.3 Inclusion and exclusion criteria

The inclusion criteria for studies were as follows: (1) inclusion of patients with multimorbidity, (2) use of observational study design, (3) report of a prediction model, and (4) death as the outcome of interest.

The exclusion criteria were as follows: (1) no development of a prediction model, (2) outcomes limited to death subgroups, (3) not written in English or Chinese, and (4) full text not retrievable despite contacting authors via email.

### 2.4 Study selection and screening

Two authors (Chen Yuanyuan and Shang Xuli) independently conducted the screening process. Initially, duplicate studies were removed, and the remaining studies were assessed based on their titles and abstracts to determine eligibility. Following the application of the inclusion and exclusion criteria, the full texts were reviewed, and the reference lists of all eligible studies were examined to identify any potentially relevant studies. In cases of disagreements regarding study selection, a discussion involving three authors (Chen Yuanyuan, Shang Xuli, and Dong Luga) was held to reach a consensus.

### 2.5 Data extraction

Two reviewers independently screened the search results. The eligibility of the full-text reports was assessed, and any discrepancies were resolved through discussion or by a third reviewer. Data from the articles selected for final inclusion were extracted using the Critical Appraisal and Data Extraction for Systematic Reviews of Prediction Modeling Studies (CHARMS) checklist. The information extracted from the selected studies was categorized into two groups: (1) Basic information, including details on the authors, year of publication, research design, participants, outcome indicator, observation time, and mortality; and (2) Model information, including information related to the prediction model, such as model development method, model validation type, variable selection method, predictive factors, model performance, calibration method, and model presentation.

### 2.6 Quality assessment

The risk of bias and the applicability of the included studies were assessed using the Prediction Model Risk of Bias Assessment Tool (PROBAST) checklist. Two authors (Chen Yuanyuan and Shang Xuli) independently evaluated the presence of bias and concerns regarding the applicability of the studies. The PROBAST checklist is utilized for the critical appraisal of studies on developing, validating, or updating prediction models for individualized predictions. It comprises 20 signaling questions categorized into four domains: participants, predictors, outcomes, and analysis. Each signaling question can be answered as “yes,” “probably yes,” “no,” “probably no,” or “no information.” If at least one signaling question in a domain is answered as “no” or “probably no,” that domain would be considered at high risk of bias. The overall risk of bias is considered low only when all domains are judged to have a low risk of bias.

### 2.7 Data analysis

Review Manager, MedCalc16.4.3 and R Studio were used for data analysis. Heterogeneity was assessed using the *I*^2^ index and Cochrane’s *Q* test. The *I*^2^ index is a measure of heterogeneity, with values of 25, 50, and 75% indicating low, moderate, and high levels of heterogeneity, respectively (25). Random- or fixed-effects models were used based on the heterogeneity of the analysis results. Additionally, Egger’s test was used to identify publication bias, with *p*-values <0.05 indicating a high likelihood of publication bias (26). A sensitivity analysis was performed using the leave-one-out method.

## 3 Results

### 3.1 Study selection

**Figure 2:**
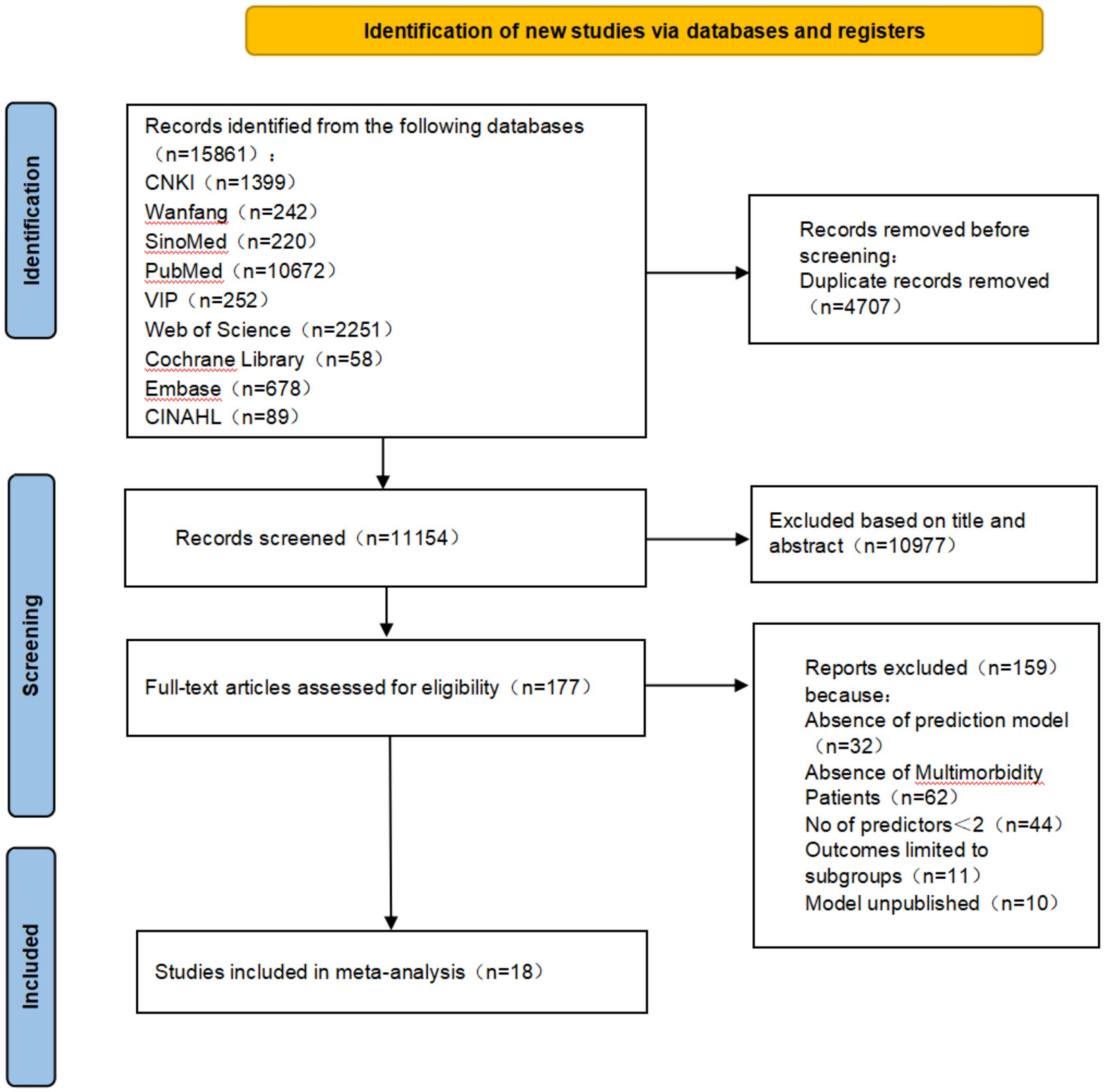
PRISMA flow diagram of the literature search and selection process.

The PRISMA 2020 guideline flowchart is shown in Figure 2. The initial database search yielded 15,861 references. After removing duplicates, 11,154 records remained across all databases. In total, 177 titles and abstracts were screened. Overall, 159 studies were excluded based on the following eligibility criteria: prediction models not established in 32 studies, inconsistent study population in 62, fewer than two predictors in 44, outcomes limited to mortality subgroups in 11, and prediction models not published in 10 studies. Ultimately, 18 studies were included in the analysis.

### 3.2 Study characteristics

Table 1 shows the characteristics of the 18 included studies. The studies were published between 2019 and 2024. Fifteen studies were conducted in China, one in the United States, one in Spain, and one in Switzerland. In addition, 15 studies were retrospective, and three were prospective. Each study was conducted in a single center. Regarding the study population, two studies focused on multimorbidity, two focused on pulmonary infection and type 2 diabetes mellitus, and the others focused on acute exacerbation of chronic obstructive pulmonary disease complicated with cerebral infarction; tuberculosis diabetes comorbidity; alcoholic cirrhosis with spontaneous bacterial peritonitis; end-stage liver disease complicated with hepatorenal syndrome; community-acquired pneumonia and type 2 diabetes mellitus; atrial fibrillation and ischemic stroke; diabetic kidney disease; chronic obstructive pulmonary disease with heart failure; cirrhosis and sepsis; coronary heart disease and chronic heart failure; obstructive sleep apnea-hypopnea syndrome with coronary heart disease; hypertension and Stanford type A aortic dissection; pulmonary tuberculosis; and secondary respiratory failure. The sample sizes ranged from 85 to 2,648 participants across the studies.

**Table 1:** Overview of basic data of the included studies.

Author/year	Country	Participants	Study design	Data source	Outcome indicator observation time	Main outcome	Mortality (%)
Wang et al. (2019) (38)	China	Elderly patients with acute exacerbation of chronic obstructive pulmonary disease AECOPD complicated with cerebral infarction	Retrospective study	One hospital	60-day admission	Mortality	(96/505)19.1
Nguyen and Graviss (2019) (39)	USA	TB treatment in patients with TB-diabetes comorbidity	Retrospective study	National Tuberculosis Surveillance System database in Texas	6 year	Mortality	(112/1227)9.1
Wang et al. (2020) (40)	China	Patients with alcoholic cirrhosis and spontaneous bacterial peritonitis	Prospective cohort study	Unknown	1 year	Mortality	(63/159)39.6
Xu (2021) (41)	China	Patients aged 18–85 years with end-stage liver disease complicated with hepatorenal syndrome	Retrospective study	One hospital	30-day admission	30-day mortality	(75/104)27.88
Cheng (2021) (42)	China	Patients 18 years of age or older with community-acquired pneumonia and type 2 diabetes mellitus	Retrospective study	Two hospitals	Duration of hospital stay	Mortality	(129/1360)9.5
Bretos-Azcona et al. (2022) (43)	Spain	Patients with high-risk multiple chronic conditions	Retrospective study	Integrated care program database	1 year	All-cause mortality	(201/591)34
Gastens et al. (2022) (28)	Switzerland	Older Adults aged 70 years and over with multimorbidity and polypharmacy	Prospective cohort study	Clinical trial	1 year	All-cause mortality	(158/805)20
Xue (2022) (44)	China	Patients older than 18 years of age with atrial fibrillation and ischemic stroke admitted to a medical ICU	Retrospective study	Medical Information Mart for Intensive Care database	30-day admission	30-day mortality	–
Han et al. (2023) (45)	China	Patients older than 60 years with type 2 diabetes mellitus complicated with pulmonary infection	Retrospective study	One hospital	Duration of hospital stay	Mortality	(28/160)17.5
Gao (2023) (46)	China	Mechanically ventilated patients aged 18 years or older with pulmonary infection and type 2 diabetes mellitus	Retrospective study	One hospital	Duration of hospital stay	Mortality	(81/403)19.95
Jin et al. (2023) (47)	China	Patients with diabetic kidney disease in the intensive care unit	Retrospective study	Medical Information Mart for Intensive Care-III, IV database	1 year	Mortality	(586/1357)43.18
Heng (2023) (48)	China	Patients with COPD and heart failure	Retrospective study	One hospital	From the day of admission to 2 weeks after discharge	Mortality	(100/1323)7.56
Xue et al. (2023) (49)	China	Patients older than 18 years with cirrhosis and sepsis	Retrospective study	One hospital	Duration of hospital stay	Mortality	(40/336)11.9
Zhang et al. (2023) (29)	China	Patients aged 18 years or older with coronary heart disease and chronic heart failure	Retrospective study	Two hospitals	3 months, 6 months, 12 months, and then every 6 months after discharge	Mortality	(220/2648)8.31
Han et al. (2024) (50)	China	Elderly patients with obstructive sleep apnea-hypopnea syndrome (OSAHS) complicated with coronary heart disease	Prospective cohort study	One hospital	1 year	Mortality	(35/250)14
Li et al. (2024) (51)	China	Patients over 60 years old with hypertension and Stanford type A aortic dissection	Retrospective study	One hospital	Duration of hospital stay	Mortality	(80/160)50
Luo et al. (2024) (52)	China	Patients aged 18 or older with severe pneumonia complicated with septic shock admitted to the emergency ICU	Retrospective study	One hospital	Duration of hospital stay	Mortality	(19/85)22.35
Fu et al. (2024) (53)	China	Patients aged 18 years or older with pulmonary tuberculosis and secondary respiratory failure	Retrospective study	One hospital	Duration of hospital stay	Mortality	(30/105)28.6

Table 2 presents detailed information on the utilized models in the 18 studies. Logistic regression was used for model development in 12 studies, Cox regression in four, a parametric Weibull regression in one, and machine learning, such as random survival forests and extreme gradient boosting, in one study. The most commonly used predictor was age, utilized in 14 studies. Other frequently used predictors included body mass index (BMI) in five studies and sequential organ failure assessment (SOFA) in three studies. The Charlson Comorbidity Index and Barthel Index were individually used in two studies.

**Table 2:** Overview of the information of the included prediction models.

Author/year	Missing data handling	Continuous variable processing method	Variable selection	Model development method	Calibration method	Validation method	Predictive factor	Model performance	Model presentation
Wang et al. (2019) (38)	Complete case analysis	Categorical variables	–	Logistic regression model	–	Internal validation	Gender, age, duration of copd, gold, nihss, cor pulmonale, respiratory failure, spontaneous pneumothorax, pulmonary infection	A:0.907 sensitivity:0.823 specificity:0.858 B:0.847 sensitivity:0.667 specificity:0.909	Normogram model
Nguyen and Graviss (2019) (39)	Complete case analysis	Categorical variables	Bayesian Modeling Averaging (BMA) method	Logistic regression model	Hosmer-Lemeshow	Internal validation	Age ≥ 65 years, being US-born, being homeless, IDU, having chronic kidney disease, TB meningitis, miliary TB, positive acid-fast bacillismear, and positive HIV status	A:0.83 (0.79–0.88) B:0.82 (0.78–0.87)	Formula of risk score obtained by partial regression coefficient of each factor
Wang et al. (2020) (40)	Complete case analysis	Categorical variables	–	Multivariate logistic regression models	ROC curve	Temporal validation	Age, Systolic blood pressure, Hypertension, Upper gastrointestinal bleeding, Hepatorenal syndrome, Maddery’s discriminant function, Albumin	A:0.805 (0.727–0.883)	ROC Curve and Formula of risk score obtained by regression coefficient of each factor
Xu (2021) (41)	Complete case analysis	Categorical variables	–	Cox regression model	Roc curve	–	International normalized ratio, hepatocellular carcinoma, percentage of neutrophils, serum creatinine	A:0.894 sensitivity:0.840 specificity:0.793	Roc curve and Kaplan–Meier curve and formula of risk score obtained by regression coefficient of each factor
Cheng (2021) (42)	–	Categorical variables	Backward stepwise selection	Multivariate logistic regression models	ROC curve and calibration plot	–	Pulse, Arterial blood PH, Age, change of consciousness, intermediate granulocyte-lymphocyte ratio, pulmonary multilobar infection, Serum sodium, Diabetic nephropathy, first fasting blood glucose	A:0.864 B:0.858	ROC Curve
Bretos-Azcona et al. (2022) (43)	Multiple imputation	Categorical variables	–	Logistic regression models	Hosmer–lemeshow	–	Bi, creatinine value, existence of pressure ulcers, and patient global status	A:0.751 (0.711–0.791) b:0.744 (0.701–0.788)	Normogram model and calculating the total risk score by summing the points for each risk factor
Gastens et al. (2022) (28)	–	Categorical variables	Backward stepwise selection	Weibull regression model	Calibration plot	–	Age, CCI, number of drugs, BMI, number of hospitalizations, BI, nursing home residency	C:0.59 (0.58–0.59)	Kaplan–Meier curve and calculating the total risk score by summing the points for each risk factor
Xue (2022) (44)	Complete case analysis	Categorical variables	LASSO regression selection	Multivariate logistic regression models	Calibration plot and ROC curve	Internal validation	Age, prothrombin time, white blood cell, blood urea nitrogen, mechanical ventilation, APSIII, SOFA	A:0.763 (0.728–0.798) B:0.748 (0.682–0.814)	Normogram model and ROC Curve
Han et al. (2023) (45)	–	Categorical variables	–	COX regression model	ROC curve and calibration plot	Internal validation	Diabetic nephropathy, Arterial blood PH, Fasting blood glucose, Neutrophil-lymphocyte ratio, Albumin, Cardiac troponin I	A:0.793 (0.747–0.829) C:0.809 B:0.797 (0.759–0.832) C:0.815	Decision Curve Analysis
Gao (2023) (46)	Multiple imputation	Categorical variables	–	Multivariate logistic regression model	Calibration plot and ROC curve	Internal validation	Age, shock, creatinine, prothrombin time	A:0.821 (0.768–0.874)	Decision Curve Analysis and normogram model
Jin et al. (2023) (47)	Deletion Method and Random Forest Multiple Imputation	Categorical variables	Bidirectional stepwise method	COX proportional hazards model	Brier score, Calibration curve, ROC curve	External validation	Age, weight, sepsis, heart rate, temperature, CCI, SAPS II, and SOFA, lymphocytes, RDW, serum albumin, metformin	A:0.771 (0.746–0.795) B:0.795 (0.756–0.834)	Normogram model
Heng (2023) (48)	Complete case analysis	Categorical variables	–	Multivariate logistic regression model	Calibration plot and Hosmer-Lemeshow	Internal validation	Age, smoking duration, copd duration, respiratory failure, nt-probnp, procalcitonin, albumin, d-dimer	C:0.874 (0.838, 0.910)	Model performance chart (roc curve)
Xue et al. (2023) (49)	Complete case analysis	Categorical variables	–	COX proportional hazards model	Calibration plot	Internal validation	Age, hepatic encephalopathy, hepatorenal syndrome, hypersensitive C-reactive protein, modified MEWS and prothrombin time	C:0.857 (0.815–0.920)	Normogram model
Zhang et al. (2023) (29)	Imputation method	Categorical variables	–	Machine learning: COX model, RSF and xgboost	–	–	Age, creatinine, N-terminal pro-brain natriuretic peptide, red blood cells, systolic blood pressure, ejection fraction, BMI, low-density lipoprotein cholesterol, urea nitrogen, triglyceride, potassium, high-density lipoprotein cholesterol, serum total cholesterol, alanine aminotransferase, blood glucose, serum total bilirubin, central nervous system disease, uric acid, diabetes mellitus, statins	C:0.902 (0.900–0.915)	SHAP model
Han et al. (2024) (50)	–	Categorical variables	–	Multivariate logistic regression model	Hosmer–Lemeshow and roc curve	Internal validation	Bmi, apnea-hypopnea index, serum high-sensitivity c-reactive protein, sleep average oxygen saturation	A:0.958 (0.926–0.980) sensitivity:0.829 specificity:0.0.963 b:0.932 (0.893–0.960) sensitivity:0.0.857 specificity:0.884	Normogram model
Li et al. (2024) (51)	Complete case analysis	Categorical variables	Stepwise regression selection	Logistic regression model	Hosmer–Lemeshow and roc curve	External validation	Aortic regurgitation, abnormal aortic contour, abdominal vascular involvement, time from onset to visit, d-dimer, left ventricular ejection fraction, systolic blood pressure, mean arterial pressure	A:0.878 (0.816–0.892) sensitivity:0.884 specificity:0.0.853 b:0.830 (0.815–0.874) sensitivity:0.908 specificity:0.0.907	–
Luo et al. (2024) (52)	Complete case analysis	Categorical variables	–	Binary Logistic Regression	ROC curve	–	Age, BMI, serum creatinine, prothrombin time,24 h fluid infusion volume	A:0.812 sensitivity:0.860 specificity:0.714	Formula of risk score obtained by partial regression coefficient of each factor
Fu et al. (2024) (53)	Complete case analysis	Categorical variables	Backward stepwise selection	Multivariate logistic regression model	ROC curve	Internal validation	Pao2/fio2, Albumin, APACHE II score, neutrophil/lymphocyte ratio, C-reactive protein	A:0.856 (0.803–0.921) sensitivity:0.826 specificity:0.755	Formula of risk score obtained by partial regression coefficient of each factor

“–,” not reported; A, development cohort; B, validation cohort; C, C-index.

GOLD, Global initiative for chronic obstructive lung disease; NIHSS, National institute of health stroke scale; TB, Tuberculosis; IDU, Injecting-drug user; BI, Barthel index; CCI, Charlson comorbidity index; BMI, Body mass index; APSIII, Acute physiology score III; SOFA, Sequential organ failure assessment; SAPS, Simplified acute physiology score; RDW, Red cell distribution width; MEWS, Modified early warning score; APACHE, Acute physiology and chronic health evaluation; AUC, Area under the curve; ROC, Receiver operating characteristic curve; RSF, Random survival forest; XGBoost, eXtreme gradient boosting.

Model performance was reported in all studies. The area under the receiver operating characteristic curve (AUC) or C-statistic values were the most frequently used indices for evaluating discrimination performance, ranging between 0.700 and 0.907. Calibration was performed in nine models, with the Hosmer–Lemeshow test being the most frequently used method.

### 3.3 Model validation

Most models in the 18 studies were internally validated, 13 of which reported 15 internal validations. Machine learning was used to develop and validate the prediction models.

### 3.4 Results of quality assessment

All the studies were evaluated as having a high overall risk of bias according to the PROBAST checklist. The assessments of the risk of bias and applicability for all the studies are summarized in Table 3. Eleven studies were judged as having a high risk of bias, indicating methodological concerns in the models’ development or validation processes.

**Table 3:** PROBAST results of the included studies.

Author/year	ROB				Applicability			Overall	
	Participants	Predictors	Outcome	Analysis	Participants	Predictors	Outcome	ROB	Applicability
Wang et al. (2019) (38)	(+)	(+)	(−)	(−)	(−)	(+)	(−)	(−)	(−)
Nguyen and Graviss (2019) (39)	(+)	(+)	(−)	(−)	?	(−)	(−)	(−)	?
Wang et al. (2020) (40)	(−)	(+)	(−)	?	(−)	(+)	(+)	?	(+)
Xu (2021) (41)	(−)	(+)	(−)	(−)	(+)	(+)	(−)	(−)	(+)
Cheng (2021) (42)	(+)	(−)	(−)	?	(+)	(−)	(−)	?	(+)
Bretos-Azcona et al. (2022) (43)	(−)	(−)	(+)	(−)	(+)	(+)	(+)	(−)	(−)
Gastens et al. (2022) (28)	(−)	(+)	(−)	(−)	(+)	(+)	(−)	(−)	(+)
Xue (2022) (44)	(−)	(−)	(−)	(−)	(−)	(−)	(−)	(−)	(+)
Han et al. (2023) (45)	(+)	(+)	(−)	?	(+)	(+)	(−)	?	(+)
Gao (2023) (46)	(+)	(+)	(−)	(+)	(+)	(+)	(−)	(+)	(+)
Jin et al. (2023) (47)	(+)	(+)	(+)	(+)	(+)	(+)	(+)	(+)	(−)
Heng (2023) (48)	(+)	(+)	(−)	(−)	(+)	(+)	(−)	(−)	(+)
Xue et al. (2023) (49)	(+)	(+)	(−)	(−)	(+)	(+)	(−)	(−)	(+)
Zhang et al. (2023) (29)	(−)	(−)	(−)	(+)	(+)	(−)	(−)	(−)	(+)
Han et al. (2024) (50)	(−)	(+)	(−)	?	(−)	(+)	(−)	?	(+)
Li et al. (2024) (51)	(+)	(−)	(−)	(−)	(−)	(+)	(+)	(−)	(+)
Luo et al. (2024) (52)	(+)	(−)	(−)	(−)	(+)	(−)	(−)	(−)	(+)
Fu et al. (2024) (53)	(+)	(+)	(−)	(−)	(+)	(+)	(−)	(−)	(+)

PROBAST, Prediction model Risk of Bias Assessment Tool; ROB = risk of bias.

+ indicates low ROB/low concern regarding applicability; − indicates high ROB/High concern regarding application; ? indicates unclear. ROB/unclear concern regarding applicability.

Six studies primarily had a high risk of bias in the participant domain, mainly attributed to the use of inappropriate data sources. Seven studies were rated as having a high risk of bias in the predictor domain because the outcome data were mainly known before assessments, and the predictors were not similarly defined or assessed for all participants.

Sixteen studies had a high risk of bias in the outcome domain, primarily owing to the lack of a pre-specified or standard outcome, blind determination between the outcome and predictors, and inappropriate time intervals between predictor assessment and outcome determination.

Eleven studies were considered to have a high risk of bias and four studies an unclear risk of bias in the analysis domain. Thirteen studies had insufficient sample sizes, with the participant numbers being <20 events per variable. Ten studies involved partial conversion of continuous variables into categorical variables. Eleven studies did not include all enrolled participants in the analysis, and missing data were inappropriately handled. Sixteen studies did not avoid the selection of predictors based on univariate analysis. Complexities in the data were not accounted for in 13 studies, and this was not reported in four. The predictive performance of the models was not reported in seven studies. Five studies did not comprehensively consider the overfitting, underfitting, and optimism performances of the models, and this was not reported in only one study. Correspondence between the predictors and their assigned weights in the final model and multivariate analysis results were not reported in four studies.

Regarding the assessment of applicability concerns, two studies were rated as having low concerns, 15 as high, and one as unclear. In the participant domain, five studies had high concerns regarding the inclusion of participants aged ≥60 years, and one study had unclear concerns regarding participant inclusion. In the predictor domain, three studies involved predictor assessments based on knowledge of the outcome data. Fourteen studies had high concerns regarding the time interval between predictor assessment and outcome determination.

### 3.5 Meta-analysis of validation models

**Figure 3:**
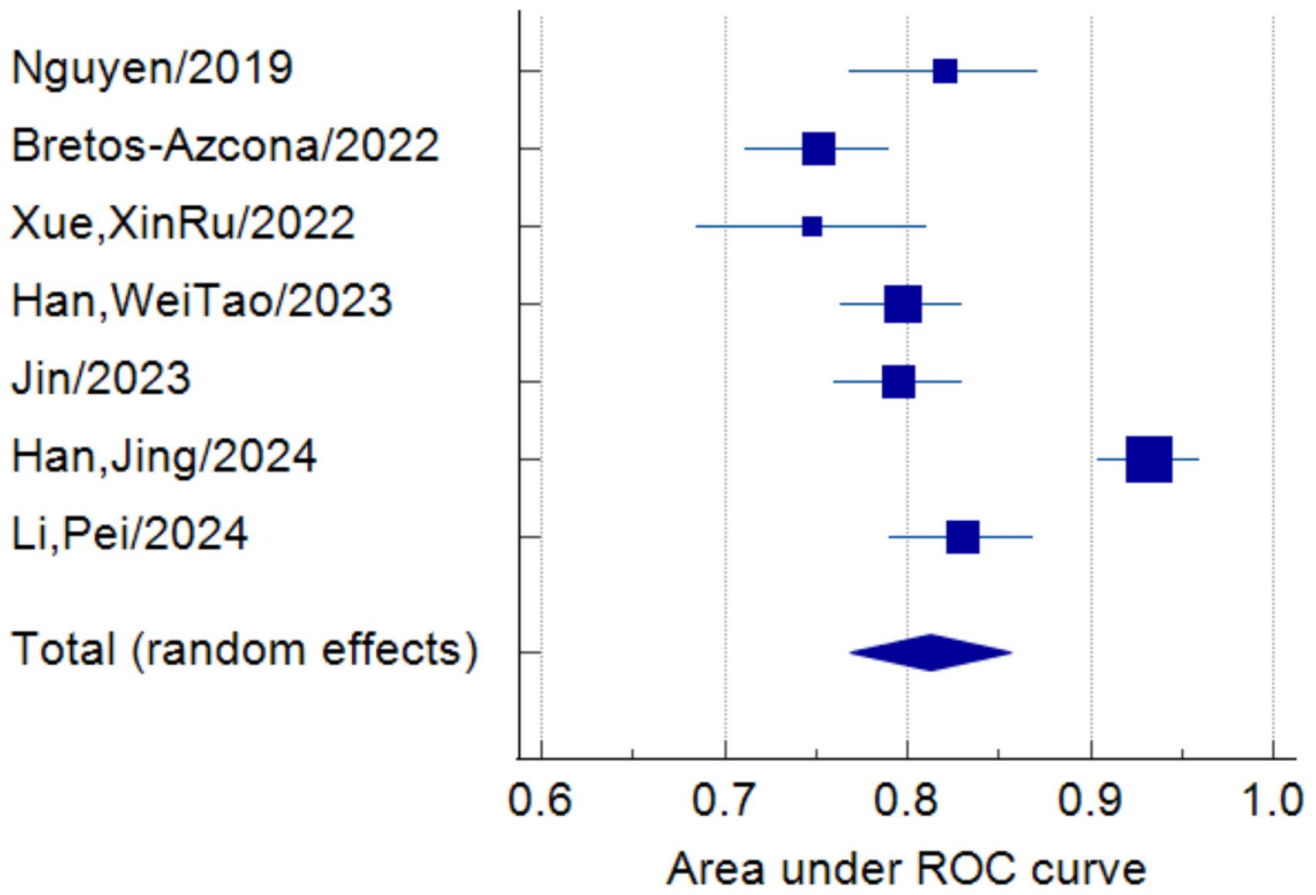
Forest plot of the random effects meta-analysis of pooled AUC estimates for seven validation models.

**Figure 4:**
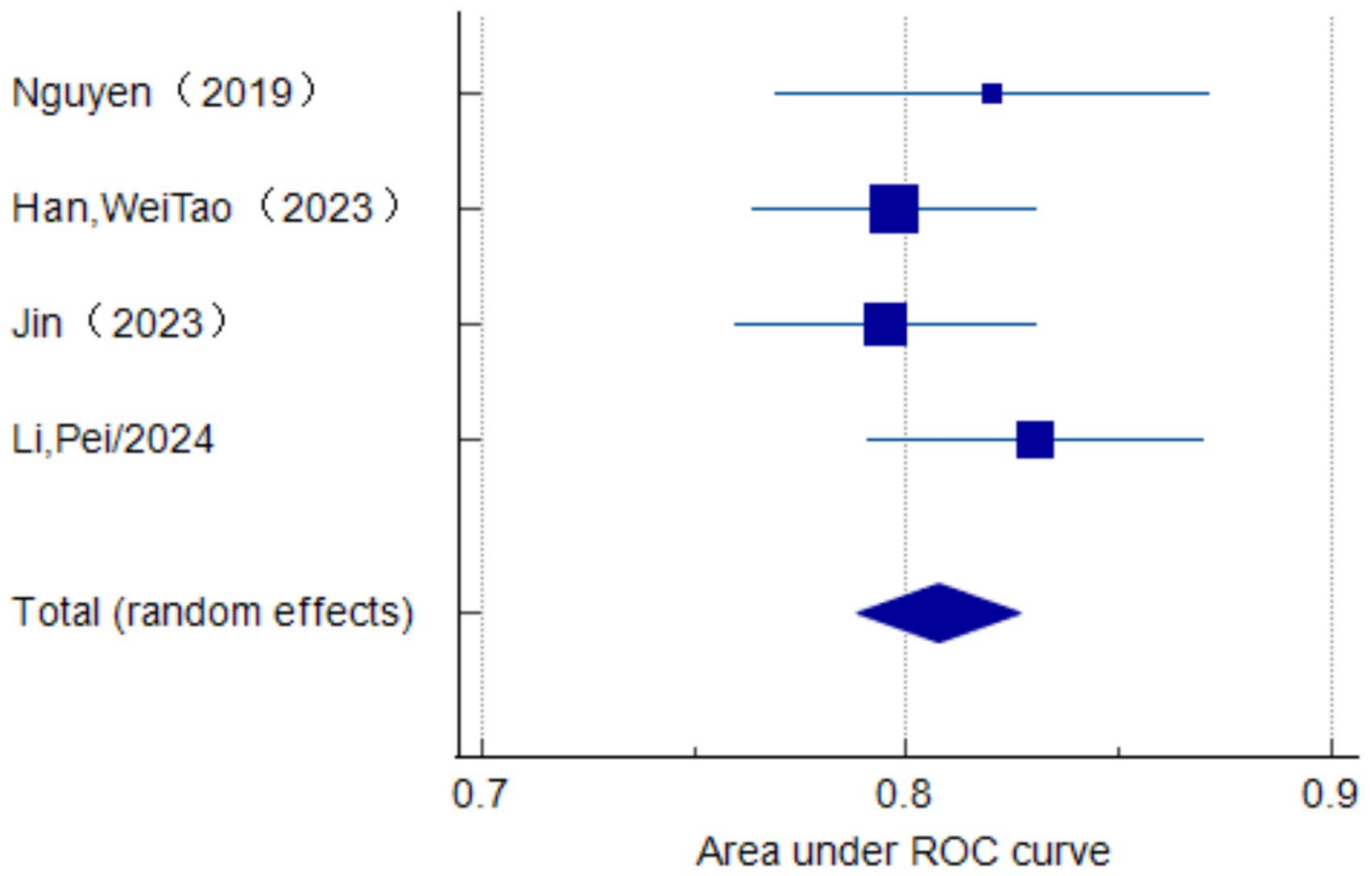
Forest plot of the random effects meta-analysis of pooled AUC estimates for the mortality outcome of four validation models.

**Figure 5:**
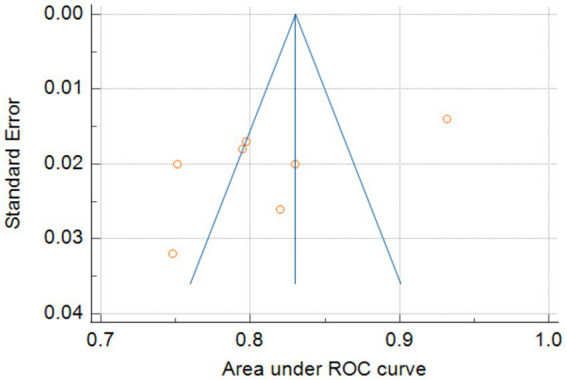
Egger’s test of seven models.

**Figure 6:**
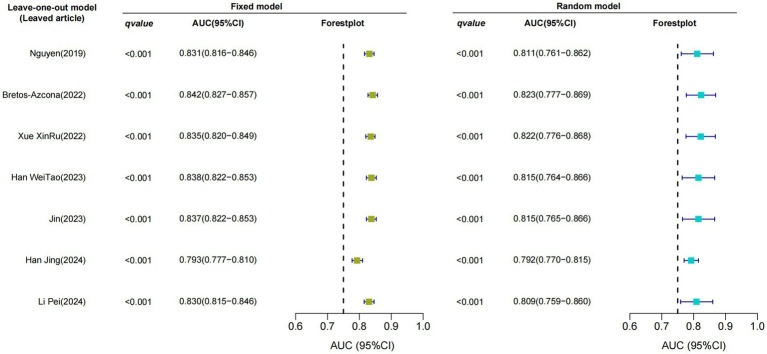
Sensitivity analysis using the leave-one-out model.

Only seven studies were eligible for synthesis owing to insufficient details on the models. A random-effects model was used to calculate the pooled AUC of seven studies, which resulted in a value of 0.81 (95% confidence interval, 0.77–0.86) (Figure 3). The *I*^2^ value was 92.77% (*p* < 0.001), indicating a high degree of heterogeneity. For models that used mortality, the pooled AUC was 0.81 (95% confidence interval, 0.79–0.83; Figure 4). The *I*^2^ value for the models was 0.0% (*p* = 0.501), indicating a low degree of heterogeneity. The Egger’s test of seven studies showed no significant publication bias, with a value of −9.39 (*p* = 0.122; Figure 5). The leave-one-out analysis showed relatively stable overall results when each study was sequentially excluded and the meta-analysis results were reconstructed. The results demonstrated good diagnostic value (AUC > 0.75; Figure 6).

## 4 Discussion

This systematic review revealed an increasing number of risk prediction models for mortality in patients with multimorbidity; however, most models used Chinese patient data. We evaluated the internal research or external validation of models from 18 studies, which demonstrated moderate-to-good predictive performance, with AUC values ranging from 0.70–0.91. However, the risk of bias was judged as high in 11 studies and unknown in four according to the PROBAST checklist, limiting the practical utility of the models. The meta-analysis revealed a pooled AUC value of the seven validated models of 0.81 (95% confidence interval: 0.77–0.86). However, heterogeneity was high in these models, which may be attributed to differences in predictors, population, and methodology. In addition, we observed inadequate reporting in adherence to the Transparent Reporting of a Multivariate Prediction Model for Individual Prognosis or Diagnosis (TRIPOD) statement in some articles (27) during model evaluation. This opacity introduces doubts and possible prejudice hazards into the models. Future researchers should prioritize the development of novel models that incorporate larger sample sizes, adhere to rigorous research designs, undergo multicenter external validation, and exhibit enhanced reporting transparency.

Valuable insights can be obtained from the development processes of the included models. For example, Gastens et al. (28) used a prospective cohort study design with a large sample size; however, they did not appropriately handle missing data and continuous and categorical predictors, which have often been neglected by other studies. In contrast, Zhang et al. (29) used a large sample size but a retrospective design, resulting in a high risk of bias. The data were sourced from the Electronic Medical Record System, with a degree of bias risk in the participant, predictor, and outcome PROBAST domains. However, their study excelled in the analysis domain. The multiple imputation method was employed to handle missing data, and comprehensive evaluations of model calibration and discrimination were conducted, which were frequently overlooked in other models. Zhang et al. (29) used a machine-learning method during model development. Machine learning methods tend to yield higher accuracy than logistic regression (30). Most included studies had issues with continuous variable treatment, sample size, and predictor selection. Some of these challenges can be addressed by incorporating machine learning into model development. However, a drawback of machine learning lies in the current lack of suitable presentation tools. Therefore, researchers must select a suitable model development method that considers the current circumstances. Generally, all models exhibited moderate or good performance; however, the risk of bias remained high. Improvements regarding the number of events, data source (case–control or cohort study), continuous variable treatment, data complexity, predictor selection method, and model calibration and fitting are required.

The existing predictor models reported in this review had some clinical implications. Initially, predictors with high frequency were demonstrated to possess a certain reference significance for future research. Furthermore, age was consistently identified as an important risk factor for mortality and multimorbidity (31, 32). The incidence of mortality increases for individuals aged ≥80 years, accounting for approximately 82% of mortality cases (33). Therefore, nurses should remain alert regarding mortality risk in patients aged ≥80 years with multimorbidity. BMI was well documented as a predictor in the models (34); however, some studies revealed certain limitations regarding using BMI for diseases such as acute heart failure in hospitalized patients or patients with coronary heart disease after a meal (35, 36). Additionally, if BMI is to be clinically utilized for assessing mortality in patients with multimorbidity, the corresponding disease-adjusted BMI cutoff value requires further exploration. In addition, SOFA is widely used in clinical practice as a disease assessment scale, demonstrating excellent predictive performance in many diseases; however, it does not directly evaluate mortality risk in patients with multimorbidity. Disease severity assessed using SOFA is associated with mortality in patients with multimorbidity (37).

### 4.1 Limitations

This review had some limitations. First, most included models were from studies conducted in mainland China, which may limit the generalizability of the findings to Western countries. Adjustments may be required when applying the models to different populations. Future researchers should develop risk prediction models for mortality in patients with multimorbidity from diverse populations. Second, a risk-of-bias tool for machine learning models has not yet been published, and the PROBAST tool may be unsuitable for assessing all included models. Third, only studies published in English and Chinese were included, and issues related to language barriers or gray literature may have been omitted. Fourth, model impact studies were not considered. Determining whether a model can be implemented into clinical practice is important and should be investigated in future research. Fourth, only seven validated models from seven studies were included in our study owing to differences in reporting methodology and transparency. This may have resulted in problems related to the sources of study heterogeneity, which could not be discussed further. Sixth, the publication bias test exhibited low statistical power. However, these problems did not impact the evaluation of the models and partly mirrored the identified methodological and reporting problems. Therefore, more transparent and rigorous methodologies are required. Moreover, the comorbid diseases and primary outcomes of the model in each article were not entirely identical, which could potentially affect the results. However, we conducted in-depth sensitivity analyses, and the findings demonstrate that our conclusions are reliable.

### 4.2 Conclusion

This systematic review revealed a pooled AUC value of seven validated models of 0.81 (95% confidence interval: 0.77–0.86), suggesting a certain level of discrimination. However, according to the PROBAST checklist, 11 included studies had a high risk of bias, and 15 studies raised concerns about their applicability in clinical practice. Most current prediction models for mortality in patients with multimorbidity do not meet the PROBAST standards. Researchers must familiarize themselves with the PROBAST checklist and strictly adhere to the TRIPOD statement to improve the quality of future research. Moreover, they should prioritize the development of new models that incorporate rigorous study designs and multicenter external validation. Our findings provide valuable insights on prediction models for clinical practice and future research, which may contribute to the development of global strategies to address this significant public health challenge.

## Data Availability

The original contributions presented in the study are included in the article/supplementary material, further inquiries can be directed to the corresponding author.

## References

[1] Ho ISS Azcoaga-Lorenzo A Akbari A Black C Davies J Hodgins P . Examining variation in the measurement of multimorbidity in research: a systematic review of 566 studies. Lancet Public Health. (2021) 6:e587–97. doi: 10.1016/S2468-2667(21)00107-9, PMID: 34166630

[2] Zöller B Connors JM. Multimorbidity, comorbidity, frailty, and venous thromboembolism. Haematologica. (2024) 109:3852–9. doi: 10.3324/haematol.2023.284579, PMID: 39618295 PMC11609784

[3] Harrison C Fortin M van den Akker M Mair F Calderon-Larranaga A Boland F . Comorbidity versus multimorbidity: why it matters. UK: London: SAGE Publications Sage (2021). 10.1177/2633556521993993 PMC7930649 33718251

[4] Xu X Mishra GD Jones M. Evidence on multimorbidity from definition to intervention: an overview of systematic reviews. Ageing Res Rev. (2017) 37:53–68. doi: 10.1016/j.arr.2017.05.003, PMID: 28511964

[5] Chowdhury SR Chandra Das D Sunna TC Beyene J Hossain A. Global and regional prevalence of multimorbidity in the adult population in community settings: a systematic review and meta-analysis. EClinicalMedicine. (2023) 57:101860. doi: 10.1016/j.eclinm.2023.101860, PMID: 36864977 PMC9971315

[6] Marengoni A Angleman S Melis R Mangialasche F Karp A Garmen A . Aging with multimorbidity: a systematic review of the literature. Ageing Res Rev. (2011) 10:430–9. doi: 10.1016/j.arr.2011.03.003, PMID: 21402176

[7] Zhou Y Dai X Ni Y Zeng Q Cheng Y Carrillo-Larco RM . Interventions and management on multimorbidity: an overview of systematic reviews. Ageing Res Rev. (2023) 87:101901. doi: 10.1016/j.arr.2023.101901, PMID: 36905961

[8] Department of Health UK (2015). Available online at: https://www.gov.uk/government/publications/living-well-for-longer-progress-1-year-on (accessed March 18, 2019)

[9] Menotti A Mulder I Nissinen A Giampaoli S Feskens EJ Kromhout D. Prevalence of morbidity and multimorbidity in elderly male populations and their impact on 10-year all-cause mortality: the FINE study (Finland, Italy, Netherlands, elderly). J Clin Epidemiol. (2001) 54:680–6. doi: 10.1016/S0895-4356(00)00368-1, PMID: 11438408

[10] Jani BD Hanlon P Nicholl BI McQueenie R Gallacher KI Lee D . Relationship between multimorbidity, demographic factors and mortality: findings from the UK biobank cohort. BMC Med. (2019) 17:74. doi: 10.1186/s12916-019-1305-x, PMID: 30967141 PMC6456941

[11] Ryan BL Allen B Zwarenstein M Stewart M Glazier RH Fortin M . Multimorbidity and mortality in Ontario, Canada: a population-based retrospective cohort study. J Comorb. (2020) 10:2235042X20950598. doi: 10.1177/2235042X20950598, PMID: 32923405 PMC7457707

[12] Nunes BP Flores TR Mielke GI Thumé E Facchini LA. Multimorbidity and mortality in older adults: a systematic review and meta-analysis. Arch Gerontol Geriatr. (2016) 67:130–8. doi: 10.1016/j.archger.2016.07.008 27500661

[13] Brown R Thorsteinsson E. Comorbidity: what is it and why is it important? In: Brown R Thorsteinsson E, editors. Comorbidity. Cham: Palgrave Macmillan (2020)

[14] Takeuchi S Kohno T Goda A Shiraishi Y Kawana M Saji M . Multimorbidity, guideline-directed medical therapies, and associated outcomes among hospitalized heart failure patients. ESC Heart Fail. (2022) 9:2500–10. doi: 10.1002/ehf2.13954, PMID: 35561100 PMC9288806

[15] Muth C Glasziou PP. Guideline recommended treatments in complex patients with multimorbidity. BMJ. (2015) 351:h5145. doi: 10.1136/bmj.h5145, PMID: 26431846

[16] Schneider C Aubert CE Del Giovane C Donzé JD Gastens V Bauer DC . Comparison of 6 mortality risk scores for prediction of 1-year mortality risk in older adults with multimorbidity. JAMA Netw Open. (2022) 5:e2223911. doi: 10.1001/jamanetworkopen.2022.23911, PMID: 35895059 PMC9331084

[17] Damen JAA Moons KGM van Smeden M Hooft L. How to conduct a systematic review and meta-analysis of prognostic model studies. Clin Microbiol Infect. (2023) 29:434–40. doi: 10.1016/j.cmi.2022.07.019, PMID: 35934199 PMC9351211

[18] Xu Z Zhang J Zhang Q Xuan Q Yip P. A comorbidity knowledge-aware model for disease prognostic prediction. IEEE Trans Cybern. (2021) 52:9809–19. doi: 10.1109/TCYB.2021.3070227 33961578

[19] Bao Y Lu P Wang M Zhang X Song A Gu X . Exploring multimorbidity profiles in middle-aged inpatients: a network-based comparative study of China and the United Kingdom. BMC Med. (2023) 21:495. doi: 10.1186/s12916-023-03204-y, PMID: 38093264 PMC10720230

[20] Fan J Sun Z Yu C Guo Y Pei P Yang L . Multimorbidity patterns and association with mortality in 0.5 million Chinese adults. Chin Med J. (2022) 135:648–57. doi: 10.1097/CM9.0000000000001985, PMID: 35191418 PMC9276333

[21] Li FR Wang S Li X Cheng ZY Jin C Mo CB . Multimorbidity and mortality among older patients with coronary heart disease in Shenzhen. China J Geriatr Cardiol. (2024) 21:81–9. doi: 10.26599/1671-5411.2024.01.005, PMID: 38440336 PMC10908585

[22] Singh M Gulati R Lewis BR Zhou Z Alkhouli M Friedman P . Multimorbidity and mortality models to predict complications following percutaneous coronary interventions. Circ Cardiovasc Interv. (2022) 15:e011540. doi: 10.1161/CIRCINTERVENTIONS.121.011540, PMID: 35861796

[23] Kato D Kawachi I Saito J Kondo N. Complex multimorbidity and mortality in Japan: a prospective propensity-matched cohort study. BMJ Open. (2021) 11:e046749. doi: 10.1136/bmjopen-2020-046749, PMID: 34341044 PMC8330573

[24] Anthonimuthu DJ Hejlesen O Zwisler AO Udsen FW. Application of machine learning in multimorbidity research: protocol for a scoping review. JMIR Res Protoc. (2024) 13:e53761. doi: 10.2196/53761, PMID: 38767948 PMC11148516

[25] Higgins JPT Thompson SG Deeks JJ Altman DG. Measuring inconsistency in meta-analyses. BMJ. (2003) 327:557–60. doi: 10.1136/bmj.327.7414.557, PMID: 12958120 PMC192859

[26] Egger M Davey Smith G Schneider M Minder C. Bias in meta-analysis detected by a simple, graphical test. BMJ. (1997) 315:629–34. doi: 10.1136/bmj.315.7109.629, PMID: 9310563 PMC2127453

[27] Collins GS Reitsma JB Altman DG Moons KGM. Transparent reporting of a multivariable prediction model for individual prognosis or diagnosis (TRIPOD): the TRIPOD statement. BMJ. (2015) 350:g7594. doi: 10.1136/bmj.g7594, PMID: 25569120

[28] Gastens V Chiolero A Anker D Schneider C Feller M Bauer DC . Development and validation of a new prognostic index for mortality risk in multimorbid adults. PLoS One. (2022) 17:e0271923. doi: 10.1371/journal.pone.0271923, PMID: 35930547 PMC9355209

[29] Zhang WC Tian J Yang H Han QH Zhang YB. 5-year all-cause mortality survival analysis and interpretable study in patients with coronary artery disease combined with chronic heart failure. Chin J Dis Control Prev. (2023) 27:391. doi: 10.16462/j.cnki.zhjbkz.2023.04.001

[30] Churpek MM Yuen TC Winslow C Meltzer DO Kattan MW Edelson DP. Multicenter comparison of machine learning methods and conventional regression for predicting clinical deterioration on the wards. Crit Care Med. (2016) 44:368–74. doi: 10.1097/CCM.0000000000001571, PMID: 26771782 PMC4736499

[31] Svensson T Saito E Svensson AK Melander O Orho-Melander M Mimura M . Association of sleep duration with all- and major-cause mortality among adults in Japan, China, Singapore, and Korea. JAMA Netw Open. (2021) 4:e2122837. doi: 10.1001/jamanetworkopen.2021.22837, PMID: 34477853 PMC8417759

[32] Hanlon P Nicholl BI Jani BD Lee D McQueenie R Mair FS. Frailty and pre-frailty in middle-aged and older adults and its association with multimorbidity and mortality: a prospective analysis of 493 737 UK biobank participants. Lancet Public Health. (2018) 3:e323–32. doi: 10.1016/S2468-2667(18)30091-4, PMID: 29908859 PMC6028743

[33] Su Z Huang L Zhu J Cui S. Effects of multimorbidity coexistence on the risk of mortality in the older adult population in China. Front Public Health. (2023) 11:1110876. doi: 10.3389/fpubh.2023.1110876, PMID: 37089511 PMC10113675

[34] Hussin NM Shahar S Din NC Singh DKA Chin AV Razali R . Incidence and predictors of multimorbidity among a multiethnic population in Malaysia: a community-based longitudinal study. Aging Clin Exp Res. (2019) 31:215–24. doi: 10.1007/s40520-018-1007-9, PMID: 30062670

[35] Yoshihisa A Sato T Kajimoto K Sato N Takeishi Y. Acute decompensated heart failure syndromes (ATTEND) investigators. Heterogeneous impact of body mass index on in-hospital mortality in acute heart failure syndromes: an analysis from the ATTEND registry. Eur Heart J Acute Cardiovasc Care. (2019) 8:589–98. doi: 10.1177/2048872617703061, PMID: 28361568

[36] Zhang YN Pang YX Chen TL Gu GQ Xie RQ Cui W. The clinical observation of postprandial hypotension in patients with obesity or overweight and coronary heart disease. J Hebei Med Univ. (2019) 40:144–8. doi: 10.3969/j.issn.1007-3205.2019.02.006

[37] Qin W Zhang X Yang L Li Y Yang S Li X . Predictive value of the sequential organ failure assessment (SOFA) score for prognosis in patients with severe acute ischemic stroke: a retrospective study. J Int Med Res. (2020) 48:300060520950103. doi: 10.1177/0300060520950103, PMID: 32865055 PMC7469749

[38] Wang WJ Huang ZD Li GY Luo YJ. Establishment of a nomogram model for short-term mortality risk in elderly patients with acute exacerbation of COPD complicated by cerebral infarction. J Pract Med. (2019) 35:2128–32. doi: 10.3969/j.issn.1006-5725.2019.13.022

[39] Nguyen DT Graviss EA. Development and validation of a risk score to predict mortality during TB treatment in patients with TB-diabetes comorbidity. BMC Infect Dis. (2019) 19:10. doi: 10.1186/s12879-018-3632-5, PMID: 30611208 PMC6321653

[40] Wang RR Jiang YY Wang XB Yang JX. A prognostic model for long-term survival in alcoholic liver cirrhosis patients complicated with spontaneous bacterial peritonitis. Hepatoday. (2020) 25:1042. doi: 10.3969/j.issn.1008-1704.2020.10.007

[41] Xu T. Establishment of death risk model for patients with hepatorenal syndrome in end-stage liver disease and relationship between TCM syndromes and laboratory indexes. Chengdu, China: Chengdu Univ TCM (2021).

[42] Cheng SJ. Risk factors analysis and prediction models establishment of in-hospital mortality among community-acquired pneumonia patients with type 2 diabetes mellitus. Liaoning, China: China Med. University (2021).

[43] Bretos-Azcona PE Ibarrola Guillén C Sánchez-Iriso E Cabasés Hita JM Gorricho J Librero LJ. Multisystem chronic illness prognostication in non-oncologic integrated care. BMJ Support Palliat Care. (2022) 12:e112–9. doi: 10.1136/bmjspcare-2019-002055, PMID: 32581004

[44] Xue XR. Development of a 30-day mortality risk prediction model in ischemic stroke patients with atrial fibrillation. Chongqing, China: Chongqing Med. University (2022).

[45] Han WT Feng M Yang QM Hu H Xu RH. Prognostic risk factors for elderly patients with T2DM and pulmonary infection. P Prev Med. (2023) 30:1373–7. doi: 10.3969/j.issn.1006-3110.2023.11.023

[46] Gao JX. Risk factors and prediction models for in-hospital death in patients with pulmonary infections and type 2 diabetes requiring mechanical ventilation. Beijing, China: Peking Union Med. College (2023).

[47] Jin W Jin H Su X Che M Wang Q Gu L . Development and validation of the prediction model for mortality in patients with diabetic kidney disease in intensive care unit: a study based on medical information Mart for intensive care. Ren Fail. (2023) 45:2257808. doi: 10.1080/0886022X.2023.2257808, PMID: 37724537 PMC10512753

[48] Heng X. Nomogram modeling of short-term mortality risk in patients with COPD and heart failure comorbidity. Luzhou, China: Southwest Med Universidad (2023).

[49] Xue LL Li BH Liu CY Li WK Chang LX Liu L. Establishment and evaluation of a multivariate cox proportional hazards prediction model for mortality during short-term hospitalization in patients with liver cirrhosis and sepsis. J Clin Hepatol. (2023) 39:1089–97. doi: 10.3969/j.issn.1001-5256.2023.05.014

[50] Han J Zhu W Jiang F. Construction of nomogram prediction model for poor prognosis of elderly patients with obstructive sleep apnea hypopnea syndrome and coronary heart disease based on multicenter. J Clin Exp Med. (2024) 23:462–5. doi: 10.3969/j.issn.1671-4695.2024.05.004

[51] Li P Xue WT Zhao P. Relationship between postoperative blood pressure control level and in hospital death in elderly patients with hypertension complicated with Stanford type a aortic dissection. Chin Heart J. (2024) 36:294–301. doi: 10.12125/j.chj.202308002

[52] Luo SP Liu DX Wei ZJ Dong WX Liu SL Qu HR . Multivariate analysis and risk model of ICU death in patients with severe pneumonia and septic shock. J Clin Intern Med. (2024) 41:104–6. doi: 10.3969/j.issn.1001-9057.2024.02.009

[53] Fu LN Rui LM Peng JH Wang HQ. Study on the construction of death risk prediction model for pulmonary tuberculosis patients with respiratory failure. Electron J Emerg Infect Dis. (2024) 9:26–30. doi: 10.19871/j.cnki.xfcrbzz.2024.01.006

